# Antimicrobial Activity of Coronarin D and Its Synergistic Potential with Antibiotics

**DOI:** 10.1155/2014/581985

**Published:** 2014-05-15

**Authors:** Nanthawan Reuk-ngam, Nitirat Chimnoi, Nisachon Khunnawutmanotham, Supanna Techasakul

**Affiliations:** ^1^Laboratory of Organic Synthesis, Chulabhorn Research Institute, Vibhavadi-Rangsit Highway, Laksi, Bangkok 10210, Thailand; ^2^Laboratory of Natural Products, Chulabhorn Research Institute, Vibhavadi-Rangsit Highway, Laksi, Bangkok 10210, Thailand; ^3^Department of Chemistry and Center of Excellence for Innovation in Chemistry, Faculty of Science, Kasetsart University, Bangkok 10900, Thailand

## Abstract

Coronarin D is a labdane-type diterpene from the rhizomes of *Hedychium coronarium*. In the view of our ongoing effort to explore its novel biological activity, antimicrobial activity study of coronarin D was performed. The results showed that coronarin D was active against tested Gram-positive bacteria, inactive for tested Gram-negative bacteria, and weakly active against tested fungi. The antibacterial effect of the combination of coronarin D with nine classical antibiotics against four Gram-positive bacteria was also evaluated. The fractional inhibitory concentration indices (FIC_I_) of coronarin D-antibiotics combinations, calculated from the checkerboard assay, were used as synergism indicator. Out of 36 combinations, 47% showed total synergism, 33% had partial synergistic interaction, 17% showed no effect, and 3% showed antagonism. By combination with coronarin D at concentration of 0.25 minimal inhibitory concentration (MIC), the activities of antibiotics were boosted to 4- to 128-fold. These finding suggested an attractive approach to combat the infectious diseases by using coronarin D-antibiotic drug combination.

## 1. Introduction

During the past to present, infectious diseases are the leading cause of death worldwide particularly in developing countries [1]. The problems are from unrecognized emerging infections, reemerging infections, more virulent pathogens, and drug-resistance bacterial infections. The infectious microorganisms that cause major public health problems can be divided into three main groups; those are opportunistic pathogens, nosocomial pathogens, and gastrointestinal pathogens. Opportunistic pathogens are infectious microorganisms that are potentially harmful for immunodeficiency or immunosuppressed patients [2]. Nosocomial pathogens are infectious microorganisms that cause problem to patients admitted to hospital for a long time [3]. For example,* P. aeruginosa* is a leading Gram-negative opportunistic and nosocomial pathogen that is seriously problematic for patients in ICUs [4].* S. aureus* and* S. epidermidis *are Gram-positive pathogenic bacteria that cause common nosocomial infection [3, 5].* A. niger* is the common opportunistic and nosocomial species associated with invasive pulmonary aspergillosis in human [6]. Gastrointestinal pathogens are infectious microorganisms that cause gastrointestinal disease such as diarrhea, typhoid fever, and cholera [7].* B. cereus *is one of gastrointestinal pathogens usually found in canned food industries and causing serious foodborne disease [8]. Nowadays, the infectious diseases become more dangerous and more infections become resistant to classical antibiotics. Therefore, the continuing efforts to search for new antibacterial substances are necessary and urgently needed. Natural products still remained as important source in drug discovery, providing crucial and unmatched chemical diversity. Most of antibiotics used today are derived from natural products or natural product scaffolds [9].

In this study, the pathogens we selected are those commonly found and those that lead to the major health problems. The selected pathogens comprised of* P. aeruginosa*,* C. albicans*,* C. albidus*,* Acremonium *sp.,* A. flavus*,* A. niger*, and* Penicillium *sp. as opportunistic pathogens,* S. aureus*,* S. epidermidis*,* P. aeruginosa*,* E. faecalis*, and* A. niger* as nosocomial pathogens, and* E. coli*,* S. typhimurium*, and* B. cereus* as gastrointestinal pathogens.

**Figure 1:**
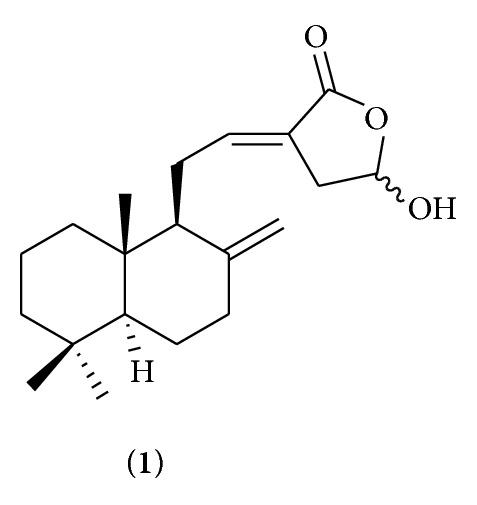
Structure of coronarin D (**1**).

Coronarin D (**1**) (Figure 1) is a labdane-type diterpene isolated mainly from the rhizomes of* Hedychium coronarium *[10, 11], which is known in Thai as “Mahahong.” Various biological activities of coronarin D were observed, for example, cytotoxic activity against cancer cell [10, 11] and inhibiting both constitutive and inducible nuclear factor-kappa B pathway, a key mediator of inflammation, apoptosis, invasion, and osteoclastogenesis [12]. Recently, antifungal activity of coronarin D against* Candida albicans *has just been reported [13]. This encouraged coronarin D to be of interest for studying its activity against different microorganisms.

For many years until now, combination therapy with two or more antibiotics is used in special cases to prevent or delay the emergence of resistant strain, to treat emergency case during the process of diagnosis, and to take advantage of antibiotic synergism [14]. Recently, the combinations of natural products and antibiotics were studied and reported to enhance the activity of classical antibiotics [15–17]. From data above, the synergistic effects between natural products and standard antibiotics were an alternative potential approach to treat infectious diseases. In the view of our ongoing effort to search for promising antimicrobial agents and explore the novel biological activity of coronarin D, we decided to study antimicrobial activity as well as synergistic effects of coronarin D to classical antibiotics.

## 2. Materials and Methods

### 2.1. Coronarin D Isolation

Coronarin D was isolated from the rhizomes of* Hedychium coronarium* according to the previous report [11] and its structure was confirmed by the spectroscopic methods (^1^H and ^13^C NMR, MS, and FTIR).

### 2.2. Tested Microorganisms

Microorganisms used for antimicrobial testing were obtained from the culture collection center, Thailand Institute of Scientific and Technological Research (TISTR), Thailand, as follows.

*Gram-Negative Bacteria. Pseudomonas aeruginosa *(*P. aeruginosa*) TISTR 781 (ATCC 9027),* Escherichia coli *(*E. coli*) TISTR 780 (ATCC 8739), and* Salmonella typhimunium* (*S. typhimunium*) TISTR 292 (ATCC 13311). 

*Gram-Positive Bacteria. Staphylococcus aureus *(*S. aureus*) TISTR 1466 (ATCC 6538),* Staphylococcus epidermidis *(*S. epidermidis*) TISTR 518 (ATCC 14990),* Enterococcus faecalis *(*E. faecalis*) TISTR 379 (ATCC 19433), and* Bacillus cereus *(*B. cereus*) TISTR 687 (ATCC 11778). 

*Yeast. Candida albicans *(*C. albicans*) TISTR 5779 (ATCC 10231) and* Cryptococcus albidus *(*C. albidus*) TISTR 5684 (MUCL 40661). 

*Fungi*.* Acremonium sp.* TISTR 3487 (MUCL 40768),* Penicillium sp*. TISTR 3118, isolated from yam-like plant (*Dioscorea hispida* Dennst), Thailand,* Aspergillus flavus* (*A. flavus*) TISTR 3366, isolated from compost, and* Aspergillus sp*. TISIR 3105, isolated from hair pomade. The latter three strains were isolated by dilution plating technique and identified by morphological characterization.

The bacteria were maintained on nutrient agar (NA) at 37°C and fungi were maintained on potato dextrose agar (PDA) at 28°C.

### 2.3. Preparation of Inoculum

The tested bacteria were cultured in nutrient broth (NB) and incubated for 18–24 h at 37°C. The tested yeast,* C. albicans* and* C. albidus*, were made by growing on PDA or Sabouraud dextrose agar (SDA) for 48 hours at 28°C. The colonies were harvested, suspended in sterile saline, and their concentrations were adjusted to a 0.5 McFarland standard, the equivalence of 1-2 × 10^8^ cfu/mL. The samples were then diluted 1 : 10,000 in Muller Hinton broth (MHB) or Sabouraud dextrose broth (SDB) to 1 × 10^4^ cfu/mL. For fungi,* Penicillium* sp.,* A. flavus*,* Aspergillus *sp.,* Acremonium *sp., and* A. niger*, spore suspension were adjusted from 0.4 × 10^4^ to 5 × 10^4^ spore/mL in sterile saline.

### 2.4. Antimicrobial Agents

Gentamicin (CN), ciprofloxacin (CIP), oxacillin (OX), amphotericin B powder (AMB), penicillin G (PNG), chloramphenicol (CRP), tetracycline (TTC), and erythromycin (ERY) were purchased from Sigma Chemical Co. (St Louis, MO). Polymyxin B (PMB) and rifampicin (RIF) were obtained from EMD Chemicals, Inc. (San Diego, CA.). Antimicrobial agents were prepared as stock solutions at concentrations of 4 and 16 mg/mL in dimethylsulfoxide for susceptibility testing and checkerboard method, respectively.

### 2.5. Susceptibility Testing

Antimicrobial activity of coronarin D was assessed by determination of the minimal inhibitory concentration (MIC), minimal fungicidal concentration (MFC), and minimal bactericidal concentration (MBC) in accordance with NCCLS guideline M38-P, M27-A3, and M7-A6 for testing of conidium-forming filamentous fungi, yeast, and bacteria, respectively [18–20]. 

*MICs.* The minimal inhibitory concentration (MIC) was determined using the twofold broth microdilution method in accordance with NCCLS guideline [20]. Concentrations of coronarin D from 0.39 to 200 *μ*g/mL were used. After incubating for additional 24 h for bacteria or 48–72 h for fungi, the lowest concentration of compound that inhibited the growth of organism was considered as MIC. 

*MBCs.* Subcultures were made by spreading visually clear broth dilution MIC well to agar media; Muller Hinton agar (MHA) for bacteria, SDA for conidium-forming filamentous fungi, and PDA for yeast, for 24 h for bacteria or 48–72 h for fungi. The lowest concentration at which there was no growth of organism was considered as MBC and MFC. Antimicrobials are usually regarded as bactericidal if the MBC or MFC value is not more than four times of the MIC value [21].

### 2.6. Checkerboard Method

The synergism or antagonism of the combinations was performed by the checkerboard technique. Standard powder forms of PNG, OX, PMB, CIP, RIF, CRP, TTC, CN, and ERY were stored at 2 to 8°C until use. The stock solutions and serial twofold dilutions of each drug or compound to at least double of the MIC value were prepared according to the recommendations of NCCLS immediately prior to testing [22]. The antibiotics used in combination were serially diluted along the ordinate, while the compound was diluted along the abscissa. Each well was inoculated with 0.1 mL of 10^5^ cfu/mL culture of test microorganism and then incubated for 24 h at 37°C for bacteria or 48 h at 30°C for fungi. Interaction was assessed algebraically by determining the fractional inhibitory concentration (FIC). The ΣFIC were calculated as follows: ΣFIC = FICA + FICB, when FICA is the MIC of drug A in the combination/MIC of drug A alone and FICB is the MIC of compound in combination/MIC of compound alone. The combination is considered as synergistic when the FIC is ≤0.5, as partial synergistic when 0.5 < FIC ≤ 0.75, as no effect when 0.75 < FIC ≤ 2, and as antagonistic when ΣFIC is >2 [23].

## 3. Results

The antimicrobial activity of coronarin D was tested in vitro against microorganisms by using broth microdilution technique and were compared with antibiotics. All MIC and MBC values of coronarin D and antibiotics were summarized in Table 1. Coronarin D could inhibit Gram-positive bacteria (*S. aureus*,* S. epidermidis*,* E. faecalis*, and* B. cereus*) at concentrations ranging from 6.25 to 50 *μ*g/mL but showed no activity against Gram-negative bacteria (*E. coli*,* P. aeruginosa*, and* S. typhimurium*) with MIC values over 200 *μ*g/mL. Coronarin D showed noticeable activity against* B. cereus *at MIC value of 6.25 *μ*g/mL, which was lower than that of oxacillin antibiotic. For* S. aureus *and,* S. epidermidis*, coronarin D also displayed moderate activity at MIC values of 12.5 *μ*g/mL. The MBC values of coronarin D against the tested Gram-positive bacteria ranged from 12.5 to 100 *μ*g/mL. From the result in Table 1, the MBC values of coronarin D against the studied Gram-positive bacteria was less than four times of its MIC values; it was therefore considered as bactericidal effect of coronarin D.

Table 2 summarized minimal inhibitory concentration (MIC) and minimal fungicidal concentration (MFC) values of antibiotic amphotericin B and coronarin D against the tested fungi. Coronarin D inhibited yeast (*C. albicans *and* C. albidus*) at concentration ranging from 25 to 50 *μ*g/mL and inhibited fungi (*A. niger*,* A. flavus*,* Aspergillus *sp.,* Acremonium *sp., and* Penicillium *sp.) at MIC values ranging from 100 to 200 *μ*g/mL. Coronarin D showed low activity for inhibition of the growth of* Aspergillus *sp. with MIC value higher than 200 *μ*g/mL. For* A. niger*,* Aspergillus *sp., and* Penicillium *sp., the MFC values of coronarin D were over 200 *μ*g/mL whereas MFC value at 200 *μ*g/mL was observed in* A. flavus *and* Acremonium *sp.

To evaluate the synergistic effects of coronarin D and antibiotics, the checkerboard assay was employed. The fractional inhibitory concentration index (FIC_I_) value was utilized to assess the synergism (total synergism, FIC_I_ ≤ 0.5; partial synergism, 0.5 < FIC_I_ ≤ 0.75; no effect, 0.75 < FIC_I_ ≤ 2; and antagonism FIC_I_ > 2) [23]. Synergistic effects were investigated only in Gram-positive bacteria. Antibiotics in synergistic testing included penicillin G (PNG), oxacillin (OX), polymyxin B (PMB), ciprofloxacin (CIP), rifampicin (RIF), chloramphenicol (CRP), tetracycline (TTC), gentamicin (CN), and erythromycin (ERY). Antibiotics were selected based on their mode of action.

The results of synergism indicated by FIC index were displayed in Table 3. Out of 36 combinations tested between coronarin D and nine antibiotics, 47% showed total synergism, 33% had partial synergistic interaction, 17% showed no effect, and 3% showed antagonism. Synergistic effects in all tested bacteria were observed from the combinations of coronarin D with oxacillin (CD-OX), gentamicin (CD-CN), and ciprofloxacin (CD-CIP) with FIC_I_ values ranging from 0.16 to 0.5. The best synergism was obtained from the combinations of coronarin D with oxacillin (CD-OX) and coronarin D with gentamicin (CD-CN) with FIC_I_ values ranging from 0.16 to 0.375 and the highest effect was from the CD-CN combination against* E. faecalis*. In the synergistic combination, concentration at 0.5 MIC of coronarin D could decrease the MIC values of oxacillin, ciprofloxacin, and gentamicin with a range of 16- to 260-fold. The highest lowering in MIC values of antibiotics was from the combination of CD-OX against* B. cereus* (258-fold), CD-CIP against* S. epidermidis* (260-fold) and CD-CN against* E. faecalis* (260-fold). Combinations of these antibiotics with coronarin D at concentration of 0.25 MIC could decrease their MIC values with a range of 4- to 128-fold and the highest reduction in MIC value of antibiotics was from CD-OX against* B. cereus* and CD-CN against* E. faecalis*. For polymyxin B, synergism was also observed against the tested bacteria and 4- to 64-fold lowering the drug MIC was found when it was combined with coronarin D at 0.5 MIC concentration. Synergistic effect from coronarin D-penicillin G combination was observed only against* B. cereus *with 64-fold lowering the drug MIC when it was combined with coronarin D at 0.5 MIC concentration. Finally, the combinations of coronarin D with tetracycline (CD-TTC), erythromycin (CD-ERY), chloramphenicol (CD-CRP), and rifampicin (CD-RIF) displayed only partial or no synergistic effect.

## 4. Discussion

The obtained results showed that coronarin D was active against Gram-positive bacteria but inactive against Gram-negative one. These could be rationalized by the capability of compound to cross or damage bacterial cell membrane. Gram-negative bacteria possess the outer membrane, in addition to cell wall, as the barrier to restrict the hydrophobic substances diffusion into the cell. On the other hand, the absence of the outer membrane in Gram-positive bacteria allowed the hydrophobic compounds to penetrate and/or damage cell membrane more easily [24]. Coronarin D, a labdane-type diterpene consisting of decalin ring and unsaturated lactone ring with one hydroxyl group was considered as a hydrophobic molecule; therefore, it could penetrate more easily into and interrupt the cell membrane of Gram-positive bacteria than that of Gram-negative bacteria. From Table 1, coronarin D showed remarkable activity against* B. cereus *at MIC value of 6.25 *μ*g/mL and moderate activity against* S. aureus* and* S. epidermidis *at MIC value of 12.5 *μ*g/mL. Isolated compounds that possess MIC value lower than 10 *μ*g/mL were considered to be a very promising anti-infection agent [25, 26]; therefore, coronarin D could be regarded as a candidate for anti-infection agent against* B. cereus*.

Drug synergy is well known and was used for a long time such as the herbal formulation in traditional medicine. Moreover, in the defense mechanism against infectious diseases of plants, diverse small molecules are produced. It is interesting to note that although most of these small molecules showed weak antibiotic activity, they are successful to combat infections via synergistic mechanisms [27]. Therefore the investigation of synergistic effect particularly between natural products and classical antibiotics is an alternative potentiated approach to fight microorganisms. Various combinations of plant metabolites with antibiotics displayed promising synergistic results such as combination of epigallocatechin gallate (EGCg) with various antibiotics [15], carnosic acid (benzenediolabietanediterpene)-tetracycline combination for the inhibition of MDR pumps [16], and baicalin (flavone glucuronide)-beta-lactam combination as beta-lactamase inhibitor [17]. As mentioned above, the synergistic combinations between coronarin D and nine antibiotics were carried out in this study.

Due to the low MIC value of coronarin D against Gram-positive bacteria, the synergistic combinations only on these strains were studied. As shown in Table 3, synergistic effect was observed when coronarin D was combined with standard drugs. The levels of synergy (FIC_I_ value) from high to low were as follows: 0.16 (CD-CN against* E. faecalis*), 0.19 (CD-CN against* S. epidermidis* and CD-OX against* S. epidermidis*), 0.25 (CD-CN against* S. aureus* and* E. faecalis*, CD-OX against* B. cereus,* and* S. aureus* and CN-PNG against* B. cereus*), 0.28 (CD-PMB and CD-CIP against* B. cereus*), 0.31 (CD-CIP against* E. faecalis*), 0.375 (CD-PMB against* S. aureus* and* S. epidermidis*, CD-CIP against* S. epidermidis* and CD-CN against* B. cereus*), and 0.5 (CD-CIP against* S. aureus *and CD-RIF against* E. faecalis*). Promising synergism results were found in the CD-OX, CD-CN, and CD-CIP formulations. The activity of antibiotics could be enhanced 16- to 260-fold when it was combined with coronarin D at concentration of 0.5 MIC, and the effective doses of antibiotics were downed to 4- to 128-fold when it was combined with coronarin D at concentration of 0.25 MIC. In the CD-OX, CD-CIP, CD-Tet and CD-CN formula, the MIC values of antibiotics were downed to ng/mL level. A decrease in MIC value of antibiotics due to the combination was very beneficial because (i) toxicity and/or side effects from antibiotics usage were reduced, (ii) the therapeutic cost was reduced, and (iii) the emerging of resistance strains was prevented or prolonged. Moreover, the process for developing a new drug is very expensive which is not possible in most developing countries and using a new drug also has risks of unknown side effects. Therefore, using well-known drugs in combination with herbal substances is quite elegant alternative method to combat infectious diseases.

**Figure 2:**
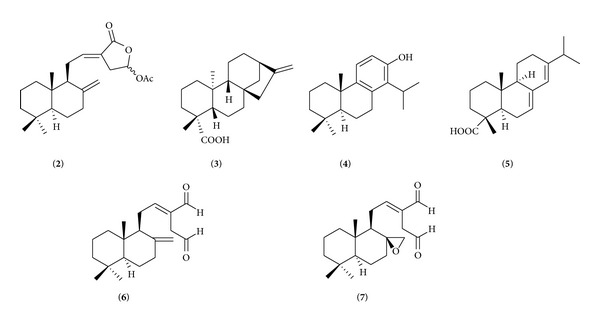
Structures of coronarin D acetate (**2**), kaurenoic acid (**3**), (+)-totarol (**4**), abietic acid (**5**), (*E*)-labda-8(17),12-diene-15,16-dial (**6**), and (*E*)-8*β*,17-epoxylabd-12-ene-15,16-dial (**7**).

Recently, in Urzúa et al.'s work [28], fifteen diterpenes were tested against* B. cereus *and* S. aureus. *They suggested two structural requirements for antimicrobial activity; those are a hydrophobic moiety and a hydrophilic region possessing one hydrogen-bond-donor group (HBD). These observations were confirmed in published reports [29, 30]. Coronarin D contains both hydrophobic part (decalin ring) and hydrophilic moiety (hydroxyl attached on lactone ring) which is fulfilled with those two requirements. In order to test the Urzúa et al.'s suggestion, coronarin D acetate (**2**) (Figure 2) was prepared. Coronarin D acetate (**2**) in which the hydroxyl group on the lactone ring was acetylated gave no activity against all tested bacteria (result from disc diffusion experiment, data not shown). This confirmed the necessity of the hydrophilic moiety in the active antimicrobial molecule.

Bacterial membranes compose of 40 percent phospholipids and 60 percent proteins [31]. The phospholipids are amphoteric molecules. It comprises two parts, that is, a polar hydrophilic glycerol containing phosphate group and a nonpolar hydrophobic fatty acid tail. In aqueous environment, it forms bilayer with the polar ends at the outmost and innermost surface and the nonpolar ends at the center of the membrane. Urzúa et al. simulated the insertion of diterpene kaurenoic acid (**3**) (Figure 2) into a phosphatidylcholine bilayer. The results revealed that kaurenoic acid (**3**) incooperated itself in the bilayer interface. The decalin ring was surrounded by hydrocarbon chains of the lipid and carboxylic group interacted with the phosphorylated group through hydrogen bonding. When kaurenoic acid (**3**) was methylated into methyl ester, the hydrogen-bond interaction to bilayer phosphorylated group was suppressed. MIC values of kaurenoic acid (**3**) were 0.16 and 0.32 *μ*g/mL against* B. cereus *and* S. aureus*, respectively, while kaurenoic acid methyl ester was inactive.

Villalaín's group studied the interactions of two diterpenes, (+)-totarol (**4**) and abietic acid (**5**) (Figure 2), against phospholipid model membrane. By using high resolution magic angle spinning-nuclear magnetic resonance (MAS-NMR), the results indicated that (+)-totarol molecule was situated in the upper region of the membrane and phenolic group was placed in the vicinity of the C-3/C4 carbon atoms of the phospholipid acyl chain [32]. Using steady-state fluorescence anisotropy measurement, it was shown that (+)-totarol (**4**) promoted the changes in physical properties of the model membranes [33]. An intrinsic fluorescent property of this molecule was also investigated in order to obtain information in location and interaction of (+)-totarol (**4**) in biomembrane model system. The results suggested that it was incorporated very efficiently into membranes and located in the inner region of the membrane far away from the phospholipid/water interface [34]. The results mentioned above demonstrated that the antibacterial action of (+)-totarol (**4**) was mediated by perturbing the membrane structure and weakening the Van der Waals interactions between the phospholipid chains. In the study of abietic acid (**5**), by using MAS-NMR, it was found that the molecule of abietic acid (**5**) was located in the upper part of the palisade structure of the membrane. The carboxyl group was in close proximity to the phospholipid ester groups and did not extend beyond C4/C7 carbons of the phospholipid molecule [35]. By using differential scanning calorimetry and ^13^P-nuclear magnetic resonance spectroscopy, it was found that abietic acid (**5**) greatly affected the phase transition of the model membrane [36]. These results clearly revealed that abietic acid (**5**) drastically changed the structural and polymorphic properties of the model membrane. In addition, the mode of action of two labdane diterpenes, (*E*)-labda-8(17),12-diene-15,16-dial (**6**) and (*E*)-8*β*,17-epoxylabd-12-ene-15,16-dial (**7**) (Figure 2), was recently investigated. The results revealed that these molecules caused significant damage and disintegration to the bacterial cell membranes and cell leakage was found [37].

(+)-Totarol (**4**), abietic acid (**5**), and coronarin D (**1**) share some structural similarities, that is, containing hydrophobic part (hydrocarbon ring) and hydrophilic moiety (phenolic, carboxylic and hydroxyl groups attached on lactone ring in (+)-totarol, abietic acid, and coronarin D, resp.). From the data mentioned above, it was anticipated that decalin ring of coronarin D (**1**) was embedded in the acyl chains of phospholipid bilayers whereas the hydroxyl group on lactone ring interacted with the phospholipid ester groups. It could be postulated that the antibacterial activity of coronarin D (**1**) may be from, at least in part, its capability to disrupt the membrane integrity and/or damage the cell membrane of Gram-positive bacteria. The mode of action in detail needs to be investigated.

## 5. Conclusion

Coronarin D (**1**) was a good antibacterial agent. It was active against Gram-positive bacteria. The promising activity was found against* B. cereus*. Synergistic effect was observed in the combination of coronarin D (**1**) to various classical antibiotics. From the literature data, the mode of action of this molecule may involve the cell membrane disruption.

## Figures and Tables

**Table 1:** Minimal inhibitory concentration (MIC) and minimal bactericidal concentration (MBC) values (*μ*g/mL) of antibiotics and coronarin D against Gram-positive and Gram-negative bacteria.

	MIC and MBC of coronarin D and antibiotics against tested bacteria (*μ*g/mL)						
	*E. coli *	*P. aeruginosa *	*S. typhimurium *	*S. aureus *	*S. epidermidis *	*E. faecalis *	*B. cereus *
MIC of coronarin D	>200	>200	>200	12.5	12.5	50	6.25
MBC of coronarin D	—	—	—	25	50	100	12.5
MIC of oxacillin	—	—	—	1.56	0.78	12.5	25
MBC of oxacillin	—	—	—	2.34	1.56	12.5	37.5
MIC of gentamicin	1.56	1.56	0.78	3.12	0.78	12.5	0.39
MBC of gentamicin	3.12	3.12	1.56	6.25	1.56	12.5	0.78
MIC of ciprofloxacin	2.34	0.39	0.39	0.195	0.39	1.56	0.39
MBC of ciprofloxacin	3.12	0.78	0.78	0.195	0.39	3.12	0.78

(—) means not test.

**Table 2:** Minimal inhibitory concentration (MIC) and minimal fungicidal concentration (MFC) values (*μ*g/mL) of amphotericin B and coronarin D against yeast and fungi.

	MIC and MFC of coronarin D and antibiotics against tested fungi (*μ*g/mL)						
	*C. albicans *	*C. albidus *	*A. niger *	*A. flavus *	*Aspergillus *sp.	*Acremonium *sp.	*Penicillium *sp.
MIC of coronarin D	50	25	125	100	200	100	150
MFC of coronarin D	200	200	>200	200	>200	200	>200
MIC of amphotericin B	0.78	0.78	1.56	3.12	3.12	6.25	6.25
MFC of amphotericin B	0.78	1.56	6.25	6.25	6.25	12.5	12.5

**Table 3:** Fractional inhibitory concentration index (FIC_I_) and minimal inhibitory concentration (MIC) of antibiotics in combination with coronarin D.

	MIC in *μ*g/mL			
	*S. aureus *	*S. epidermidis *	*E. faecalis *	*B. cereus *
Coronarin D (CD)	12.5	12.5	50	6.25
Penicillin G	0.048	0.048	0.78	100
Lowest antibiotic in combination with CD (concentration of CD, gain^a^)	0.006 (0.5MIC, 8)	0.006 (0.5MIC, 8)	1.56 (0.5MIC, 0.5)	1.56 (0.5MIC, 64)
Penicillin G + CD at 0.25MIC (gain)	0.024 (2)	0.024 (2)	1.56 (0.5)	6.25 (16)
FIC_I_^b^ (concentration of CD/concentration of antibiotic)	0.625 (6.25/0.006)	0.5625 (1.56/0.024)	2.0625 (3.12/1.56)	0.25 (0.78/12.5)

Oxacillin	1.56	0.78	12.5	25
Lowest antibiotic in combination with CD (concentration of CD, gain)	0.024 (0.5MIC, 64)	0.006 (0.5MIC, 130)	0.097 (0.5MIC, 128)	0.097 (0.5MIC, 258)
Oxacillin + CD at 0.25MIC (gain)	0.048 (32)	0.024 (32)	0.78 (16)	0.195 (128)
FIC_I_ (concentration of CD/concentration of antibiotic)	0.25 (1.56/0.097)	0.1875 (1.56/0.048)	0.25 (6.25/1.56)	0.25 (0.78/3.12)

Polymyxin B	25	50	200	50
Lowest antibiotic in combination with CD (concentration of CD, gain)	0.78 (0.5MIC, 32)	3.12 (0.5MIC, 16)	50 (0.5MIC, 4)	0.78 (0.5MIC, 64)
Polymyxin B + CD at 0.25MIC (gain)	3.12 (8)	12.5 (4)	200 (1)	1.56 (32)
FIC_I_ (concentration of CD/concentration of antibiotic)	0.375 (3.12/3.12)	0.375 (1.56/12.5)	0.75 (25/50)	0.28 (1.56/1.56)

Ciprofloxacin	0.195	0.39	1.56	0.39
Lowest antibiotic in combination with CD (concentration of CD, gain)	0.012 (0.5MIC, 16)	0.0015 (0.5MIC, 260)	0.012 (0.5MIC, 130)	0.006 (0.5MIC, 64)
Ciprofloxacin + CD at 0.25MIC (gain)	0.048 (4)	0.048 (8)	0.097 (16)	0.048 (8)
FIC_I_ (concentration of CD/concentration of antibiotic)	0.5 (3.12/0.048)	0.375 (1.56/0.097)	0.3125 (12.5/0.097)	0.3125 (0.39/0.097)

Rifampicin	0.003	0.0003	0.78	0.024
Lowest antibiotic in combination with CD (concentration of CD, gain)	0.0007 (0.5MIC, 4)	0.00015 (0.25MIC, 2)	0.024 (0.5MIC, 32)	0.024 (0.5MIC, 1)
Rifampicin + CD at 0.25MIC (gain)	0.003 (1)	0.00015 (2)	0.195 (4)	0.024 (1)
FIC_I_ (concentration of CD/concentration of antibiotic)	0.75 (6.25/0.0007)	0.75 (3.12/0.00015)	0.5 (12.5/0.195)	1.5 (3.12/0.024)

Chloramphenicol	12.5	12.5	12.5	6.25
Lowest antibiotic in combination with CD (concentration of CD, gain^a^)	12.5 (0.5MIC, 1)	3.12 (0.5MIC, 4)	6.25 (0.25MIC, 2)	0.048 (0.5MIC, 130)
Chloramphenicol + CD at 0.25MIC (gain^a^)	12.5 (1)	12.5 (1)	6.25 (2)	3.12 (2)
FIC_I_^b^ (concentration of CD/concentration of antibiotic)	1.5 (6.25/12.5)	0.75 (6.25/3.12)	0.75 (12.5/6.25)	0.5078 (3.12/0.048)

Tetracycline	0.195	0.195	0.195	0.39
Lowest antibiotic in combination with CD (concentration of CD, gain)	0.195 (0.5MIC, 1)	0.048 (0.5MIC, 4)	0.097 (0.5MIC, 2)	0.39 (0.5MIC,1)
Tetracycline + CD at 0.25MIC (gain)	0.195 (1)	0.097 (2)	0.195 (1)	0.39 (1)
FIC_I_ (concentration of CD/concentration of antibiotic)	1.5 (6.25/0.195)	0.75 (3.12/0.097)	1 (25/0.097)	1.5 (3.12/0.39)

Gentamicin	3.12	0.39	12.5	0.78
Lowest antibiotic in combination with CD (concentration of CD, gain)	0.024 (0.5MIC, 130)	0.006 (0.5MIC, 65)	0.048 (0.5MIC, 260)	0.024 (0.5MIC, 32)
Gentamicin + CD at 0.25MIC (gain)	0.195 (16)	0.012 (32)	0.097 (128)	0.195 (4)

FIC_I_ (concentration of CD/concentration of antibiotic)	0.25 (1.56/0.39)	0.1875 (1.56/0.024)	0.15625 (6.25/0.39)	0.375 (0.78/0.195)

Erythromycin	0.39	0.78	0.39	3.12
Lowest antibiotic in combination with CD (concentration of CD, gain)	0.195 (0.25MIC, 2)	0.195 (0.5MIC, 4)	0.195 (0.5MIC, 2)	0.78 (0.5MIC, 4)
Erythromycin + CD at 0.25MIC (gain)	0.195 (2)	0.78 (1)	0.39 (1)	1.56 (2)
FIC_I_ (concentration of CD/concentration of antibiotic)	0.75 (3.12/0.195)	0.75 (6.25/0.195)	1 (25/0.195)	0.75 (3.12/0.78)

^a^gain >1: decrease concentration of antibiotic, <1: increase concentration of antibiotic, and 1: no effect.

^b^The best fractional inhibitory concentration index; FIC_I_ ≤ 0.5 means total synergism, 0.5 < FIC_I_ ≤ 0.75 means partial synergism, 0.75 < FIC_I_ ≤ 2 means no effect, and FIC_I_ > 2 means antagonism.
